# Initiation of Meiotic Recombination in Mammals

**DOI:** 10.3390/genes1030521

**Published:** 2010-12-22

**Authors:** Rajeev Kumar, Bernard de Massy

**Affiliations:** Institute of Human Genetics, UPR1142, CNRS, 141 rue de la Cardonille, 34396 Montpellier cedex 5, France; E-Mail: rajeev.kumar@igh.cnrs.fr

**Keywords:** Meiosis, hotspot, DSB, crossover, recombination, *Spo11*, *Mei1*, *Mei4*, *Prdm9*

## Abstract

Meiotic recombination is initiated by the induction of programmed DNA double strand breaks (DSBs). DSB repair promotes homologous interactions and pairing and leads to the formation of crossovers (COs), which are required for the proper reductional segregation at the first meiotic division. In mammals, several hundred DSBs are generated at the beginning of meiotic prophase by the catalytic activity of SPO11. Currently it is not well understood how the frequency and timing of DSB formation and their localization are regulated. Several approaches in humans and mice have provided an extensive description of the localization of initiation events based on CO mapping, leading to the identification and characterization of preferred sites (hotspots) of initiation. This review presents the current knowledge about the proteins known to be involved in this process, the sites where initiation takes place, and the factors that control hotspot localization.

## 1. Introduction

Meiosis is an essential process whereby the number of chromosomes of diploid germ cells is halved during sexual reproduction. The production of haploid gametes from a diploid germ cell requires one round of DNA replication coupled with two successive nuclear divisions. During the first reductional division, which is a unique feature of meiosis, homologous chromosomes separate, and during the second equational division sister chromosomes are segregated. The faithful segregation of homologous chromosomes (homologs) requires specialized mechanisms to connect them, thereby, ensuring their correct orientation at metaphase I and their subsequent migration to opposite spindle poles. Meiotic recombination establishes these physical connections by forming crossovers (COs), which are reciprocal exchanges of genetic material between homologs [1]. Lack of recombination affects the accurate segregation of homologs with fatal consequences for gamete formation. Thus, failure to precisely regulate the frequency or the position of COs can cause zygotic lethality or aneuploidy, which can lead to congenital birth defects such as Down syndrome [2].

**Figure 1:**
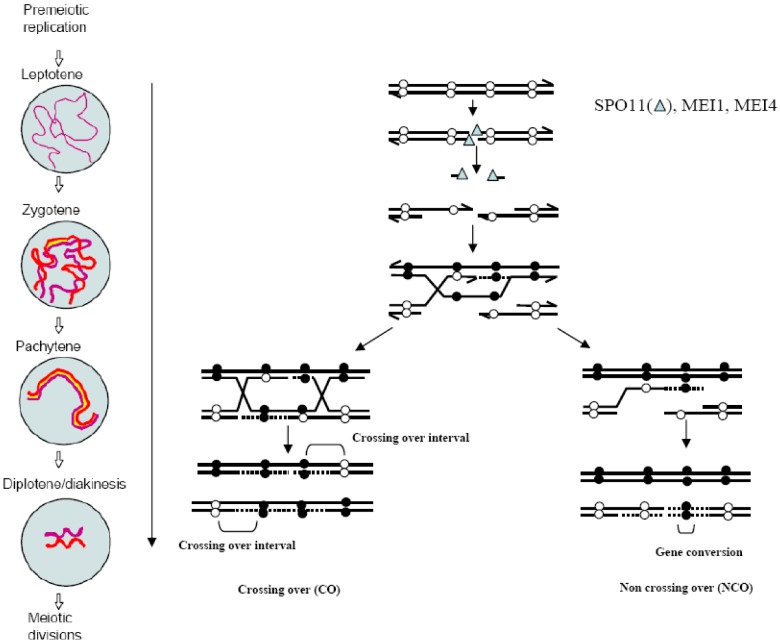
The molecular mechanism of crossovers (COs) and non-crossovers (NCOs) formation by double-strand breaks (DSB) repair: DSB formation occurs at the leptotene stage and requires SPO11, MEI4 and MEI1. DSB repair proceeds until the end of pachytene when recombinant products, either CO or NCO are formed. Black and white spheres represent polymorphisms. Meiotic stages reproduced from [14] with permission.

The molecular mechanism of meiotic recombination has been investigated in great detail in *Saccharomyces cerevisiae* and *Schizosaccharomyces pombe* [3,4] and most of its features are conserved in higher eukaryotes as well [5]. Meiotic recombination is initiated by the induction of programmed DNA double-strand breaks (DSBs). Spo11 catalyzes DSB formation via a DNA Topoisomerase II-like reaction to generate a protein-DNA intermediate in which Spo11 is covalently bound to the DNA 5' terminus through a phospho-tyrosine bond [6,7]. Spo11 is then removed by endonucleolytic cleavage that generates short Spo11-bound oligonucleotides [8]. The DNA 5' ends are then processed to produce 3' single stranded overhangs on either side of the break. One protruding 3' end searches for regions of homology and forms a displacement loop by invading an unbroken non‑sister homologous chromatid in a RAD51- and DMC1-dependent process called strand invasion [9,10]. Then, the invading 3' end provides a template for DNA synthesis and DSBs can be repaired by either synthesis-dependent strand annealing or DSB repair, leading to the formation of non-crossover (NCO) or CO products, respectively [11] (Figure 1). The formation of at least one “obligatory” CO between each homolog pair is required for proper reductional segregation. Therefore, mechanisms involved in the regulation of DSB formation and repair may, by and large, govern CO formation. How this regulation controls the precise number of COs is not yet understood [12]. 

One major open challenge concerning the initiation of meiotic recombination is to understand the biochemical mechanisms involved in DSB formation and the function of the proteins that act in concert with SPO11 to promote DSB formation. A second equally important issue is to figure out how initiation sites are selected. In this review, we will focus on the recent discoveries that have provided new insights into our understanding of meiotic recombination initiation in mammals and we will discuss the possible mechanisms of how initiation sites are chosen in the genome. 

## 2. Initiation of Meiotic Recombination by DSB in Mammals

Meiotic recombination is initiated by the induction of DSBs soon after the meiotic S phase, at leptonema, the beginning of prophase I. DSB repair takes place during the following stages (zygonema and pachynema) and is completed before diplonema and diakinesis, when homologs are connected by chiasmata (Figure 1). These steps occur at different developmental stages in male and female germ cells in mammals [13]. In females, the entire pool of oogonial cells enters meiosis synchronously during embryonic development. All recombination steps are completed before birth and oocytes are arrested at the dictyate stage. By contrast, in males, a first pool of spermatogonia enters meiosis after birth and proceeds to prophase I, from day 10 (leptonema) to day 19 (diplonema) in mice. Then, they differentiate into haploid cells during the second meiotic division and during spermiogenesis to produce mature sperm. Additional waves of meiotic differentiation of spermatogonia occur during the whole reproductive life. 

Direct molecular evidence of the initiation of meiotic recombination by DSB was first obtained in *S. cerevisiae* [15] and various approaches in *S. cerevisiae* and *S. pombe* have provided a genome-wide map of meiotic DSBs [16,17,18], although it remains to be understood how the DSB sites are selected in the genome. The direct evidence of meiosis-specific DSBs in mammals is technically more challenging than in yeast because of the heterogeneity of cells in gonads and the greater genome complexity. DNA breaks, but not necessarily only meiotic DSBs, were detected in mouse spermatocytes by PCR assay or *in situ* labeling of testis sections [19,20]. Indirect evidence of DSB formation is suggested by the high level of phosphorylation at serine 139 of the histone variant H2AX (referred as γH2AX) that is known to mark DSBs and also some other DNA lesions [21]. γH2AX is detected at leptonema and less strongly at the zygotene stage of prophase I in oocytes and spermatocytes. In mice, H2AX phosphorylation is SPO11-dependent and therefore is a convincing mark of meiotic DSB formation [22]. Furthermore, Scott Keeney’s lab showed the presence of SPO11 protein-DNA covalent complexes in mouse spermatocytes [8] and further consolidated the evidence for the conserved role of SPO11 in DSB formation. 

## 3. Proteins Required for Initiation

### 3.1. SPO11: A Universal Catalytic Inducer of Meiotic DSBs

SPO11 is an evolutionarily conserved meiotic protein present in most eukaryotic genomes [23]. The catalytic role of SPO11 in meiotic DSB was recognized because of the significant homology detected between Spo11 and the A subunit of Topoisomerase VI (Topo VI) ([6] reviewed in [24]) and from the identification of Spo11-DNA covalent complexes in *S. cerevisiae* [7]. Indeed, mechanistic models driven from structural studies of Topo VI have been instrumental in outlining the basic mechanisms of the DNA cleavage activity of SPO11 [25,26,27,28]. SPO11 has a catalytic tyrosine-containing domain, which is called the winged-helix DNA-binding domain (WHD), and a Topoisomerase/Primase (TOPRIM) domain, which harbors acidic residues needed to coordinate the binding of a magnesium ion. Although *in vitro* biochemical evidence for SPO11 catalytic activity is still lacking, a model of the mechanism of DNA cleavage by SPO11 has been proposed and reviewed in details elsewhere [29]. 

**Figure 2:**
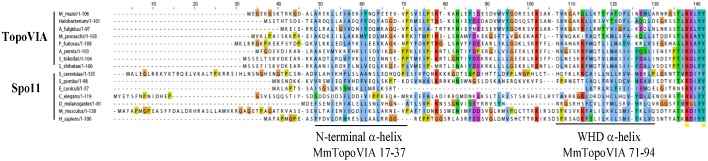
Spo11/TopoVI A orthologs: Alignment of the *N*-terminal region of TopoVIA and Spo11 orthologs, up to the conserved catalytic tyrosine. The two helixes of the TopoVIA subunit at the A-B subunit interface are underlined. Alignment provided by J. Berger, UC Berkeley.

Mammalian SPO11 orthologs were identified about a decade ago based on significant homology between mouse EST cDNAs and *S. cerevisiae Spo11* (Figure 2). Northern blot analysis revealed that mouse and human SPO11 are highly expressed in testis [30,31,32,33]. These results were confirmed by extensive RT-PCR assays in which the full-length open reading frame of *Spo11* was amplified from mouse and human testes [30,32]. In addition, transcripts that corresponded to SPO11 ESTs of the TOPRIM domain could be detected in mouse and human thymus [32]; however, the SPO11 protein, if present, is not involved in the specialized recombination events occurring in these cells since *Spo11^−/−^* mice have normal immunoglobulin class switching and somatic hypermutation [34]. RT-PCR products for the incomplete sequence of human SPO11 were also observed in several types of cancer cells from prostate, colon and ovarian carcinomas [32] and SPO11 is a known cancer testis antigen [35]. The temporal variation of *Spo11* expression was examined in the mouse during the first wave of spermatogenesis. *Spo11* was detected as early as day 7 after birth by RT-PCR analysis [30]. By Northern blotting, weak *Spo11* expression was observed at day 12 when DSBs are formed and increased levels of expression were recorded from day 17 onwards at pachytene/diplotene stage [33]. Specifically, high steady-state levels of *Spo11* were observed in pachytene spermatocytes and low levels in leptotene/zygotene spermatocytes as well as in round spermatids [33]. The developmentally regulated expression of *Spo11*in mouse testis was confirmed by *in situ* hybridization experiments in which *Spo11* transcripts were strongly detected in pachytene/diplotene spermatocytes and a weaker signal was detected in zygotene cells, secondary spermatocytes and round spermatids [30]. *Spo11* mRNA was also detected in female germ cells by *in situ* hybridization [30] and in embryonic ovaries by RT-PCR [32]. Finally, immunofluorescence studies on surface-spread spermatocyte nuclei localized SPO11 as chromatin foci in leptotene and in a discontinuous pattern in the region of homologous synapsis during zygotene and pachytene [36]. This localization of SPO11 on the synapsed chromosome axis could not be reproduced with other antibodies [37] and thus needs to be confirmed. 

Human and mouse *Spo11* cover 14.0 Kb of DNA, contain 13 exons and encode several alternative splice variants. Variation of 38 amino acids in exon 2 (isoforms with or without this exon are called beta and alpha respectively) and of four amino acids in exon 8 in both mouse and human *Spo11* [30,32] as well as a 13 amino acid insertion before exon 5 in mouse *Spo11* [31] and an 11 amino acid insertion at exon 8 in human *Spo11* have been described to produce several isoforms [32]. The functions of these isoforms are yet unknown, however, expression of *Spo11*β and *Spo11*α have been compared by quantitative PCR amplification in different types of testicular cells and in juvenile mice testes, where spermatocytes enter meiosis synchronously [38]. *Spo11*β is predominantly expressed in early spermatocytes, while *Spo11*α transcripts accumulate during late prophase I from mid-pachynema. The relatively late expression of *Spo11*α has been confirmed by the quantitative analysis of mouse mutants (*Atm^−/−^*, *Hop2^−/−^* and *Dmc1^−/−^*) that show spermatogenesis arrest at the mid-pachytene checkpoint and in which *Spo11α* is barely detectable [38]. Since SPO11 is present at low levels in mouse in testes, it can only be detected after immunoprecipitation followed by western blotting. Using such assays, the differential expression of SPO11α and β is consistent with the kinetics of *Spo11* isoforms transcripts in either wild type or mutant mice testes where spermatogenesis arrests at different stages [8,38]. SPO11 can be found covalently linked to 5' ends of DNA with a free 3' hydroxyl end in extracts from mouse testis [8]. In these complexes the DNA fragment linked to SPO11 is a short oligonucleotide, a feature that has important implications for the mechanism of SPO11 removal and the maturation of the DSB ends. Interestingly, these SPO11-oligonucleotide complexes appear to have a molecular weight compatible with the presence of *Spo11*β and are readily detected in *Dmc1^−/−^* testes where SPO11α is significantly reduced [8]. These observations support the hypothesis of distinct roles for these two SPO11 variants during meiosis and favor a role for SPO11β in generating DSBs, whereas SPO11α may have some other unknown function at mid-pachytene. Alternatively, SPO11α may have no specific function by itself or be a negative regulator of SPO11β. For instance, SPO11α could suppress SPO11 catalytic activity by dimerizing with SPO11β. It is interesting to note that exon 2, which is present only in SPO11β, overlaps with the predicted region of interaction between the TopoVI A and B subunits (Figure 2) [25,27]. However, no ortholog of the TopoVI B subunit has been identified in mammals. 

**Figure 3:**
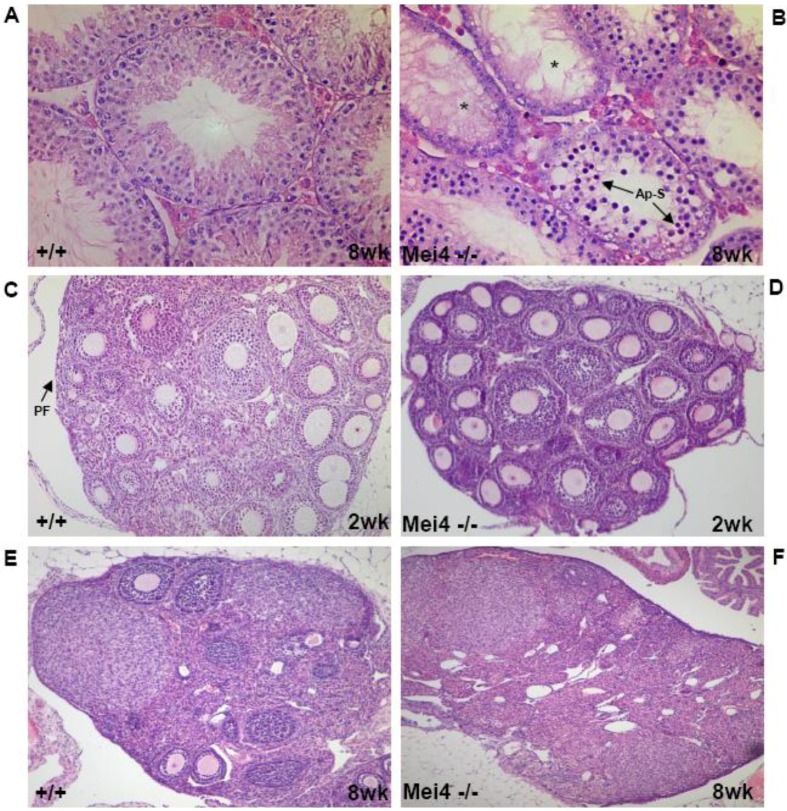
Gametogenesis arrest due to the absence of meiotic DSBs. Similar phenotypes are observed in *Spo11^−/−^*, *Mei4^−/−^* and *Mei1^−/−^*: Spermatogenesis in wild-type (**A**) and *Mei4^−/−^* (**B**) mice at 8 weeks after birth. Oogenesis in wild-type (**C**, **E**) and in *Mei^−/−^* (**D**, **F**) mice at 2 and 8 weeks after birth. Paraffin sections of paraformaldehyde-fixed tissues were stained with hematoxylin and eosin. *: Empty tubules; Ap-S: Apoptotic spermatocytes; PF: Primordial follicles.

Disruption of *Spo11* by removal of exon 4 to 6 (including the predicted catalytic tyrosine in exon 5) in the mouse causes male and female infertility [36,39]. Testes from *Spo11*^−/−^ mice are smaller and histological analysis (see Figure 3 for a similar phenotype) reveals that spermatogenesis is arrested at a stage corresponding to mid-pachynema [40] and spermatocytes are eliminated by apoptosis. No haploid cells are formed. In *Spo11^−/−^* females, a reduced (about 2-fold lower) number of oocytes complete prophase I by progressing to diplotene/dictate stage, but these mice display premature ovarian failure because of massive elimination of oocytes after birth. Comparison of oocyte loss in mutant mice that are deficient in either DSB formation (*Spo11^−/−^*) or repair (e.g., *Dmc1^−/−^*, *Atm^−/−^*) indicates that a significant fraction of *Spo11^−/−^* oocytes can survive after diplotene, whereas oocytes in which DSB repair is defective are aborted before or at dictyate arrest [41]. Hence, phenotypic differences can occur in the DNA damage-independent and -dependent responses to meiotic recombination defects in females. 

**Figure 4:**
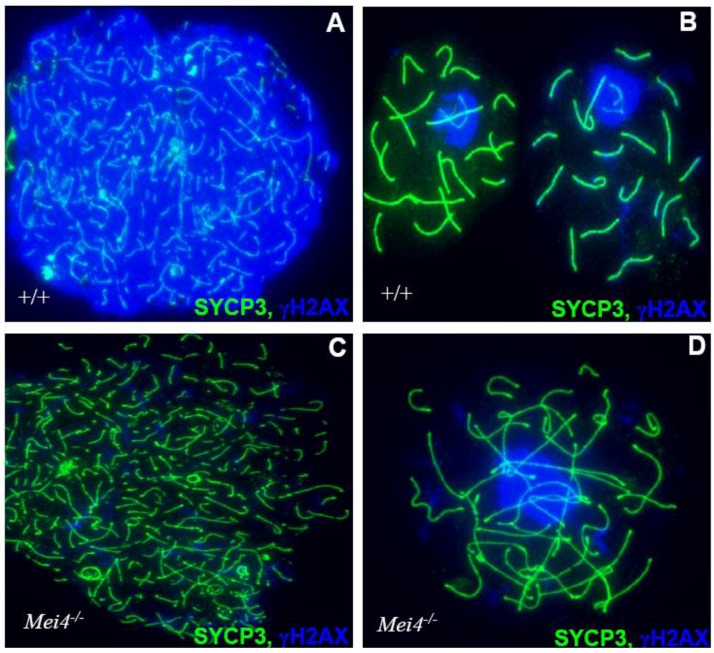
Monitoring DSB formation in spermatocyte spreads: Chromosome axes are visualized by the detection of SYCP3 (green) and DSBs by γH2AX (blue) either in wild type^(+/+)^ leptotene (**A**) and pachytene (**B**) or *Mei^−/−^* leptotene (**C**) and zygotene-like (**D**) cells. Similar phenotypes are observed in *Spo11^−/−^* and *Mei1^−/−^*.

The molecular defects due to *Spo11* inactivation in mice were assessed on surface-spread chromosomes of meiocytes by immunostaining with antibodies against proteins that are specific for DNA damage (γH2AX), DSB repair (RAD51, DMC1), axis structure (SYCP3) and synapsis (SYCP1) (see Figure 4 for a similar phenotype). In wild type mice, during meiosis, SPO11-generated DSBs trigger ATM-dependent phosphorylation of H2AX at leptonema. Once DSBs sites are processed, RAD51 and DMC1 are loaded to repair DSBs and to initiate synapsis formation between homologous chromosomes. The total number of DSBs is estimated at 250–500 based on the number of RAD51 and DMC1 foci. A lack of meiotic DSBs in *Spo11^−/−^* spermatocytes was deduced from the absence or the severely reduced level of γH2AX that marks chromatin during leptotene [22,42]. The residual γH2AX in leptotene *Spo11^−/−^* spermatocytes is thought to derive from DNA lesions that occur in a SPO11-independent fashion at or before leptonema. Conversely, some zygotene-like *Spo11^−/−^* spermatocytes accumulate γH2AX in sub-nuclear regions to form a structure called pseudo-sex body [40]. The precise mechanism of pseudo-sex body formation is unknown, but it shares some similarity with that of the sex body that normally covers the X and Y chromosomes in wild type mice, as it involves the presence of BRCA1 and ATR on unsynapsed chromosome axes [43]. The absence of RAD51/DMC1 foci in *Spo11^−/−^* meiocytes further indicates a defect in meiotic DSB formation. Moreover, a molecular assay to detect recombinant molecules at *Psmb9*, a mouse recombination hotspot, showed that in *Spo11^−/−^* ovaries COs and NCOs are not formed [44]. The absence of SPO11 also results in synapsis defects with the presence of univalents (*i.e*., unpaired chromosomes) or partially synapsed non‑homologous chromosomes [36,39]. These synapsis defects make it difficult to estimate the exact stage of meiotic arrest in *Spo11^−/−^* spermatocytes. Therefore, the expression of H1t, a testis-specific histone H1 variant, and of XMR, a member of the X-linked multigene family, has been used to determine meiotic progression in *Spo11^−/−^* spermatocytes. H1t replaces somatic H1 at mid-pachytene [45], while XMR shows dispersed staining at leptotene and later becomes associated with unsynapsed regions of the X and Y chromosomes in sex bodies [46]. H1t and XMR expression indicate that mutant spermatocytes reach a developmental stage similar to mid-pachytene [40]. Another interesting phenotype observed in *Spo11^−/−^* mice is the enrichment in cells at the bouquet stage, when telomeres cluster in a region of the nuclear periphery at the leptotene/zygotene transition, suggesting that the absence of meiotic DSBs may affect chromosome dynamics [47]. Defective DSB formation and lack of synapsis are partially rescued by treatment with cisplatin, a drug that generates DNA lesions in the genome [36]. The catalytic role of the conserved tyrosine in mouse *Spo11* was demonstrated by the observation that the defects in DSB formation and synapsis in *Spo11^Y138F/Y138F^* mice are similar to those of *Spo11*^−/−^ mutants [48]. SPO11 activity seems also to be important for ATM-mediated functions, as *Spo11*^+/−^*Atm*^−/−^ spermatocytes overcome the mid‑prophase arrest due to ATM deficiency [49] and progress to metaphase I. This rescue, which may be due to a lower level of meiotic DSBs in *Spo11*^+/−^ compared to *Spo11*^+/+^, is milder in females [50]. Altogether, evidence presented here strongly corroborates the conserved catalytic role of mouse SPO11 in meiotic DSB formation. 

### 3.2. MEI4 and REC114: Two Proteins That Are Evolutionary Conserved from Yeast to Mouse

MEI4 and REC114 were isolated in genetic screens that identified at least ten genes required for the formation of meiotic DSBs in *S. cerevisiae* [51,52]. Biochemical and cytological studies revealed that yeast *Mei4* and *Rec114* strongly interact and partially co-localize on meiotic chromosomes to form a complex that is distinct from the Spo11-complex [53,54]; see [29] for review. The precise functions of *Mei4* and *Rec114* are unknown. Moreover, because of the high degree of sequence divergence no clear ortholog of *Mei4* and *Rec114* could be identified outside closely related budding yeast species, except for *Rec7*, which is thought to be the functional ortholog of *Rec114* in *S. pombe* [55]. Recently, *Mei4* and *Rec114* orthologs in the mouse and other higher eukaryotes have been identified by focusing on short stretches of conserved sequences [56]. Primary structural alignments of all the orthologs revealed several short regions called signature sequence motifs (SSMs) with highly conserved sequence except in a few species such as *S. macrospora*, *D. melanogaster* and *C. elegans*.

The putative orthologs of *Mei4* and *Rec114* in the mouse are 4930486G11RIK and 2410076I21RIK, two proteins with unknown functions. The overall amino acid identity between the mouse orthologs and *S. cerevisiae Mei4* and *Rec114* is very low (8% and 6%, respectively). Altogether, SSMs only represent about one fifth of the whole sequence in both orthologs. Six SSMs at the *N*- and *C*-terminus are present in the *z* ortholog and may play a role in protein interaction because of the annotated alpha-helical or coiled-coil structures. Conversely, the *Rec114* ortholog has six SSMs (1 to 6) at the *N*-terminus that are predicted to adopt a beta-sheet structure and one SSM (SSM7) at the *C*-terminus that may acquire an alpha-helical form. The significance of the SSMs was recognized from the analysis of the interaction between mouse MEI4 and REC114. Indeed, like in yeast, MEI4 and REC114 strongly interact. Deletion analysis showed that this interaction requires the *N*-terminal motif (SSM1) but not the *C*-terminal region of MEI4 and both *N*- and *C*-terminal regions of REC114. Human MEI4 and REC114 also interact as monitored in a yeast two hybrid assays [57].

**Figure 5:**
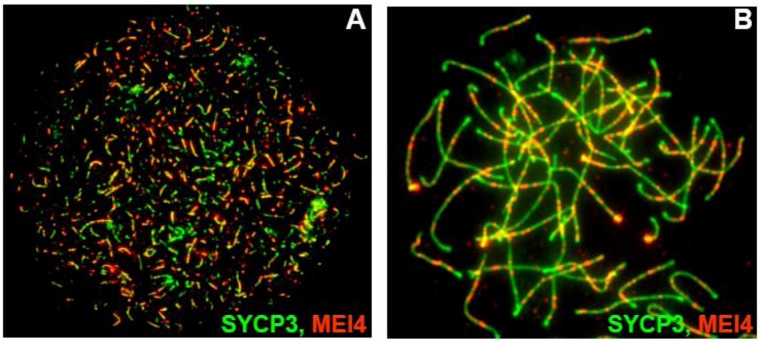
MEI4 (red) localizes as discrete foci along unsynapsed chromosome axes (labeled with SYCP3, green) at leptonema (**A**) and zygotene-like stage (**B**) in *Spo11^−/−^* spermatocytes.

*Mei4* and *Rec114* are expressed in testes and embryonic ovaries. Analysis of the steady-state levels of *Rec114* and *Mei4* transcripts during the first wave of spermatogenesis by quantitative PCR showed that their expression peaks between day 10 and 14 after birth, around the time of DSB formation and decreases thereafter. *Mei4* expression is about 100-fold higher than that of *Rec114,* but several-fold lower than that of *Spo11*. The mechanisms that regulate *Mei4* and *Rec114* expression are not known; however, the presence of an E2F6-binding element (TCCGC) upstream of the *Mei4* promoter suggests that, like other germ cell specific genes, transcription of *Mei4* may be repressed via E2F6 in somatic cells [58,59]. MEI4 has not been detected so far in testis extracts by western blotting, presumably due to its low abundance. However, the results of immunofluorescence studies in meiotic cells correlates well with the expression of *Mei4* at early prophase I. During leptotene and zygotene stages of male and female meiosis, MEI4 forms numerous foci that largely co-localize on chromosome axes and are excluded from regions of synapsis. On average the number of MEI4 foci per nucleus at leptotene is ~300, some nuclei having more than 500 foci. This number declines to ~120 foci per nucleus at zygotene and is further reduced at subsequent stages of prophase I. The number of MEI4 foci is in good agreement with the number of DSBs estimated in mouse leptotene nuclei by other means [60]. Co-labeling with antibodies against MEI4 and DMC1 and RPA (DSB repair factors) revealed that MEI4 foci do not co-localize with DMC1 and RPA, which are present at DSB sites. MEI4 localizes on chromosome axes independently of SPO11 since MEI4 foci also accumulate on unsynapsed regions of *Spo11*^−/−^ spermatocytes (Figure 5). What determines MEI4 foci localization is not known.

The conservation of *Mei4* function in DSB formation was demonstrated by analyzing *Mei4^−/−^* mice in which exon 2, which harbors the translation initiation codon and the two conserved SSM1 and SSM2 motifs, was deleted [56]. *Mei4^−/−^* male gonads are smaller and lighter; spermatogenesis is arrested at mid-prophase I and spermatocytes are eliminated by apoptosis. *Mei4*^−/−^ ovaries have a normal number of growing follicles at two weeks after birth, but they are all lost in adulthood (Figure 3). These phenotypes strongly resemble the ones described in *Spo11*^−/−^ mice. Cytological analysis of *Mei4^−/−^* spermatocytes and oocytes revealed strong reduction of γH2AX (Figure 4) and lack of RAD51, DMC1 and RPA foci, indicating that meiotic DSBs do not form in *Mei4^−/−^* mice. Immuno‑staining to investigate synaptonemal complex formation in *Mei4^−/−^* meiocytes showed nuclei with either unsynapsed chromosomes or partially synapsed non-homologous chromosomes, like in *Spo11^−/−^* meiotic cells. Formation of pseudo-sex bodies due to the absence of meiotic DSBs was also observed in a large fraction of *Mei4^−/−^* zygotene-like spermatocytes. *Mei4^−/−^* spermatocytes appear to progress to mid-pachytene before being eliminated by apotosis as suggested by the detection of the H1t variant. MEI4 may activate SPO11 directly or through other partners, thus ensuring that DSBs are formed on chromosome axes. MEI4 displacement from chromosome axes after DSB formation may provide a means to down-regulate DSB formation. Although the functional role of mouse REC114 in DSB formation remains to be elucidated, the interaction between mouse REC114 and MEI4 suggests that REC114 may also be required for meiotic DSB formation.

### 3.3. MEI1: A Protein of Unknown Function

Mouse *Mei1* (Meiosis Defective 1) was identified in a forward genetic screen that was performed with chemically mutagenized mouse embryonic cells to isolate genes involved in mouse fertility [61]. *Mei1^−/−^* mice, which harbor a frame-shift mutation that produces a truncated *Mei1* mRNA, show hypogonadism with decreased gonad weight. Male meiosis is arrested at mid-prophase I, whereas a small fraction of *Mei1^−/−^* oocytes complete the first meiotic division, but achiasmatic chromosomes do not congress to the metaphase spindle equator, thus, leading to abnormal oogenesis due to unequal chromosomal segregation [62]. The requirement of MEI1 for the initiation of meiotic recombination was demonstrated by cytological analysis of *Mei4^−/−^* meiocytes that revealed drastically reduced γH2AX at leptotene, lack of RAD51 foci and defective synapsis with small stretches of non‑homologous synapses. These phenotypes are similar to those of *Spo11^−/−^* and *Mei4^−/−^* mice and are partially rescued by cisplatin-induced DNA damage, indicating that MEI1 is essential for meiotic DSB formation [62,63]. Disruption of *Mei1* causes significant enrichment in cells at the bouquet stage, similarly to *Spo11^−/−^*, suggesting a possible role of meiotic DSB in chromosome dynamics during prophase I [47]. Several single nucleotide polymorphisms (SNPs) in the coding region of human *MEI1* have been identified, but no clear association with azoospermia in humans could be detected [64]. 

Mouse *Mei1* is thought to comprise 31 exons spanning ~55 Kb [63]. By northern blotting and RT‑PCR amplification *Mei1* transcripts were detected in mouse testes as early as five days after birth and in embryonic ovaries at E17, but not in adult ovaries, indicating that in females expression is restricted to the time of initiation of meiotic recombination [63]. Mouse *Mei1* codes for a protein predicted to be 1268 aa long. MEI1 has no apparent homolog in yeast, flies and worms, but orthologs of MEI1 have been found in mammals, chicken, zebrafish and recently in plants [63,65]. Mutations in *AtPRD1* (the *Arabidopsis thaliana* homolog of *Mei1*) cause similar defects in meiotic DSB formation to the ones observed in mouse (*i.e*., absence of early recombination markers such as DMC1 foci) [65]. Furthermore, interaction between AtPRD1 and AtSPO11-1 was revealed by yeast two-hybrid assays [65]. MEI1 orthologs contain putative ARM (Armadillo) domains with predicted similarities with Importin. Nonetheless, the biochemical function of MEI1 during meiotic DSB formation remains to be determined. 

### 3.4. WDR61 (Ski8) and MRE11/RAD50/NBS1 Are Involved in DSB Formation in *S. cerevisiae*, but Possibly not in Mammals

In budding yeast, Ski8 localizes to the nucleus during meiosis, associates with meiotic chromosomes and promotes meiotic DSB formation by directly interacting with Spo11 [66]. The role of Ski8 in Spo11 activity is conserved in several fungi [67,68], but does not seem to be universal as a mutation in the *Arabidopsis thaliana* Ski8 ortholog does not cause defects in meiotic recombination [69]. In mice, WDR61 is the homolog of yeast Ski8, but no interaction between WDR61 and SPO11 has been reported and its role in meiotic DSB formation has not yet been tested.

MRE11/RAD50/NBS1 (the MRN complex also known as Mre11/Rad50/Xrs2 in *S. cerevisiae*) is an evolutionary conserved complex that is essential for DSB repair but its role in meiotic DSB formation has only been shown in budding yeast [29]. In the mouse, the *MRN* genes are highly expressed in testis [70,71] and the MRN complex is localized in spermatocyte nuclei from the pre-leptotene to diplotene stages of prophase I [40,72]. Since the MRN complex is essential for viability in the mouse, inactivation of any of the MRN genes causes embryonic lethality, thus preventing further study of these mutant mice [73]. Therefore, mice that harbor MRN hypomorphic alleles have been used to partly delineate the function of the MRN complex during meiosis and have been reviewed in [74]. Hypomorphic *Mre11* and *Nbs1* mice show delayed meiotic progression during prophase I, incomplete and aberrant synapsis of homologous chromosomes, persistence of RAD51 foci and alterations in the frequency and localization of MLH1 foci, a marker of CO formation [75]. Overall, these studies suggest an essential role of the MRN complex in the repair of meiotic DSBs, but not in the generation of SPO11-dependent DSBs.

## 4. Sites Where Initiation Occurs

As already mentioned, direct molecular mapping of initiation sites has not been performed in mammals. The location and frequency of initiation of meiotic recombination is therefore indirectly deduced from the analysis of CO products. Upon DSB repair, exchange points are located next to the DSB site and at variable distance depending on how far the recombination intermediate extends. Thus, CO mapping provides a good estimate of the localization of initiation sites. CO frequency, even when measured at Kbp resolution, gives only an approximate idea of initiation frequency because DSB repair can yield alternative products, such as NCOs or inter-sister recombinants, in proportions that may vary in the genome [11] (Figure 1).

### 4.1. The Anatomy of a Hotspot

**Figure 6:**
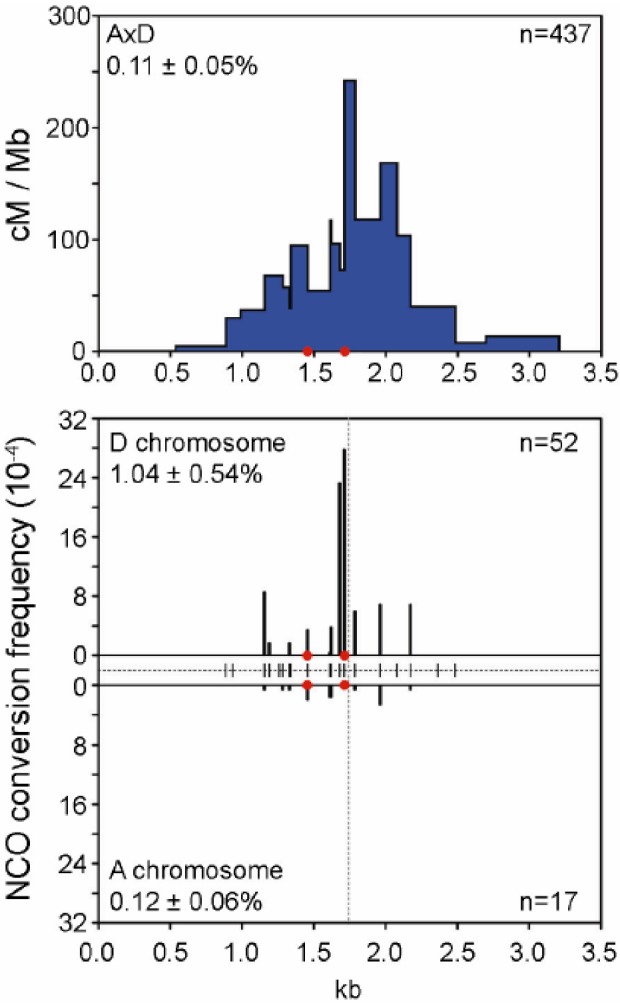
CO and NCO frequency and distribution at the mouse A3 hotspot. At this hotspot, most COs occur in a 1.5 Kb region with the highest density (cM/Mb) in the center around position 1.7 Kb (upper panel), which corresponds to the zone with the highest NCO frequency (lower panel). NCO frequencies differ on each homolog (A and D) suggesting a difference in initiation activity. Reprinted from Cole *et al*. [90] with permission.

CO events can be detected by different methods (such as direct molecular assays, pedigree or population diversity analysis) and have been shown to take place in discrete regions of the genome where initiation occurs preferentially (hotspots) [76,77,78]. Several hotspots in humans and mice were mapped at high resolution by PCR-based assays to detect recombinant molecules or through the mapping of recombination events by pedigree analysis. In a few cases, the detection of NCOs confirmed the region of initiation both in humans [79,80,81] and mice [44,82,83,84]. CO frequency is highly variable, ranging from 0.0005 cM to 2 cM. The shape of the best-fit distribution of CO at most hotspots is a narrow bell-shaped curve that is best explained by a narrow zone of initiation and limited spreading of recombination intermediates from the site of initiation over 1–2 Kb (Figure 6). In a few cases, a transmission bias of markers located near the center of the hotspot can be observed and inferred to result from differential initiation activities on each homolog both in humans [81,85,86] and mice [82,84,87,88,89,90]. In such context, the distance between the exchange points of reciprocal CO products can be measured and provides an estimate of the average size (between 220 and 540 bp) of gene conversion tracts involved in DSB repair. In contrast, NCO gene conversion tracts are much shorter, between 20 to 290 bp [80,91]. These data are consistent with the idea that COs and NCOs result from two distinct DSB repair pathways, a property largely documented in yeast [4]. In mice, functional analysis at one hotspot (*Psmb9*) indicates that NCO and CO formation occurs through different pathways that rely on Spo11 (both NCOs and COs) and MLH1/MLH3 (only COs) activity [44]. 

### 4.2. General Hotspot Properties

**Figure 7:**
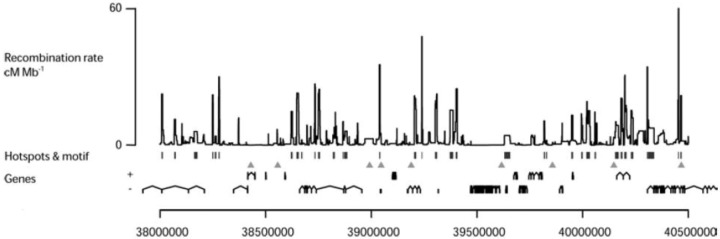
Distribution of LD-based hotspots: The estimated sex-averaged recombination rates in a 2.5 Mb region on human chromosome 21 are shown. Statistically significant hotspots are identified by vertical bars and the 7-mer motif, (CCTCCCT) which is a shorter version [94] of the 13-mer motif (see below section 4.3), by triangles. The genes in both orientations are shown [98].

Human diversity data based on linkage disequilibrium (LD) analysis [92] has provided a genome‑wide high resolution map of the predicted historical, sex averaged CO activity [93,94]. Several of the features of individual hotspots determined by direct analysis can be applied genome-wide. About 25000 LD-based hotspots have been mapped at high resolution (at intervals smaller than 5 Kb) in the human genome [94,95] using data from the Phase 2 Hapmap [96] which includes genotypes from geographically diverse populations. Overall, hotspots map at 1–2 Kb intervals, with variable estimated activities, are spaced by 50–100 Kb (Figure 7) and lie preferentially outside genes [94]. This last feature has been reported also for CO events detected by pedigree analysis in humans, supporting the conclusion that the findings derived from population diversity data reflect the property of recombination to occur outside genes rather than being the consequence of a selection effect [97]. 

### 4.3. Sequence Motifs at Hotspots

The large number of hotspots identified in the human genome allowed searching for motifs that are particularly frequent at hotspots. The most common motif is a degenerate13-mer sequence (CCNCCNTNNCCNC, hereafter called the 13-mer motif) estimated to be present in 41% of hotspots [95] and identified in some experimentally characterized hotspots such as DNA3, DMB2, DNA2 and MS32. A direct role for this motif has been proposed with the discovery that it corresponds to a high affinity binding site for the human PRDM9 protein (see below). The presence of a less degenerate motif (CCTCCCTNNCCAC, the core motif) in unique DNA leads to hotspot activity in 10% of the cases; however, this frequency increases to 73% when the core motif is associated with the LTR of the retrotransposon THE1A. Ten percent of hotspots have a core motif (perfect match) in the context of a repeat element (including those from the THE1 family, which are the most common, and also L2, AluY, AluSg and AlusX), but only 1.3% of hotspots in unique DNA have this core motif. Other motifs, such as the 9-mer CCCCACCCC, have been found to be significantly enriched at hotspots, but whether they contribute to hotspot activity is unknown [94]. Although this analysis points towards a contribution of the primary DNA sequence to hotspot activity, several experiments indicate that other factors, besides the local DNA sequence, also play an important role. For instance, the detailed study of the MSTM1a and 1b hotspots showed variations in their activities that could not be explained by polymorphisms within 100 Kb around these hotspots [86].

### 4.4. Chromatin Structure at Hotspots

**Figure 8:**
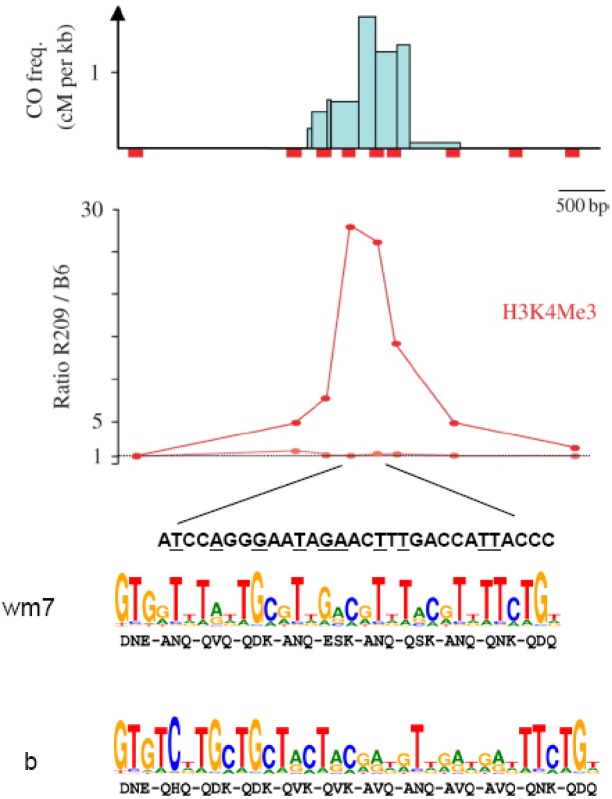
H3K4Me3 enrichment and PRDM9 binding sites at the mouse *Psmb9* hotspot: At the *Psmb9* hotspot, which is active in the *M. m. molossinus* strain R209 but not in the *M. m. domesticus* strain C57BL/6 (B6), COs are clustered in a 1.5 Kb interval as shown by the CO frequencies (cM per Kb) in this region. H3K4Me3 (measured at nine positions along the hotspot) is enriched in R209 spermatocytes in the center of the hotspot as shown by the R209/B6 ratio. A sequence with a partial match to the predicted binding site of the mouse PRDM9^wm7^ variant, which is present in the R209 strain, is found in the region with the highest CO density and with the highest H3K4Me3 enrichment. Bases matching the highest score bases from the PRDM9^wm7^ predicted motif are underlined. The PRDM9^b^ variant from B6 is predicted to recognize a distinct DNA motif (adapted from [99,105]).

The analysis of the chromatin structure and of its modifications at two mouse hotspots (*Psmb9* and *Hlx1)* has provided new clues about the specific features that may define the substrate for the initiation machinery [99]. *Psmb9* and *Hlx1* have similar activities (about 1 and 2cM respectively) but they are active only in some specific genetic backgrounds [82,84,100,101]. Analysis of chromatin modifications, particularly H3K4Me3 (trimethylation of histone H3 at lysine 4), revealed some properties shared by both hotspots and correlated with their activity [99]. H3K4Me3 enrichment is detected at the center of the hotspots, specifically in strains where the hotspots are active (Figure 8) and from chromatin extracted from whole testis or from fractions enriched for spermatocytes at the pachytene stage of meiotic prophase (when DSB are being repaired). The implication of H3K4Me3 in DSB formation is further supported by the finding that, in strains that show initiation activity at *Psmb9* on only one of the two homologs, H3K4Me3 is enriched only on the active chromosome. In addition, H3K4Me3 enrichment can be already detected on chromatin from testis of pre-puberal mice at 9 days *post partum*, when the first wave of meiotic DSB is induced [102], and it is still seen on chromatin from *Spo11^−/−^* spermatocytes where DSB are not formed. These observations indicate that H3K4Me3 marks these initiation sites before or at the time of meiotic recombination initiation and subsists during prophase progression, whereas bulk H3K4Me3 strongly decreases during prophase [99]. H4Ac5 (acetylation of H4 at lysine 5) also is enriched at the *Psmb9* and *Hlx1* hotspots. This mark may be linked to DSB repair, since H4Ac5 enrichment at the *Psmb9* hotspot is significantly reduced in *Spo11^−/−^* compared to wild type mice. Interestingly, H3K27Me3 and H3K9Me3, two modifications known to be associated with repressed chromatin status, and that have only been tested at the *Psmb9* hotspot, are depleted at the active hotspot. Conversely, the global nucleosome occupancy and chromatin accessibility at the *Psmb9* hotspot are not influenced by the activity (or non-activity) of this hotspot, as measured by H3 density and micrococcal nuclease sensitivity. This hotspot also does not contain a DNAse I hypersensitive site [103]. Complementary studies, to assess nucleosome positioning at four other mouse hotspots on chromosome 19, show that, at these hotspots, nucleosomes are well positioned and occasionally some domains are depleted in nucleosomes near the center of the hotspots. Interestingly this pattern is stable during spermatogenesis from spermatogonia to pachytene [104]. 

### 4.5. The Role of *Prdm9* in Hotspot Specification

**Figure 9:**
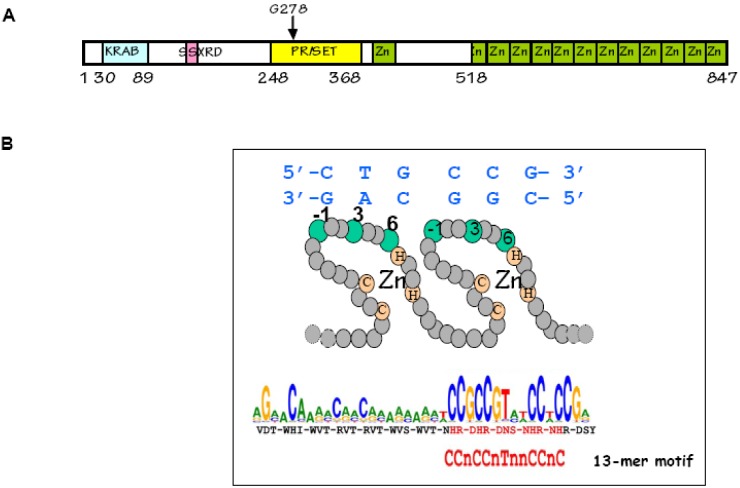
The PRDM9 protein: (**A**) PRDM9 contains several known protein domains: KRAB, SSXRD, PR/SET and C2-H2 Zinc fingers. The residue G278 in mouse PRDM9 is essential for its methyltransferase activity [109]. The zinc finger array is part of a single exon; (**B**) In each zinc finger, three residues (−1, 3 and 6) are in contact with the DNA (residue 2 plays a minor role and is in contact with the opposite strand). The sequence predicted to be recognized by the major variant of human PRDM9 is shown and overlaps with the 13-mer motif [95].

In a number of cases it has been reported that a given hotspot can show variations in its recombination activity among individuals in humans and strains in mice [81,82,85,86,89,97,100,106,107]. The conclusion, that at least some of these effects could not be accounted for by local differences in DNA sequences, raised the possibility that long distance effects or *trans-*acting factors might be involved in the regulation of hotspot activity. Genetic studies in mice showed that a *trans‑*acting factor was indeed involved [100,108] and the gene responsible for this effect was proposed to be *Prdm9* [99]. The PRDM9 protein (also called MEISETZ) contains a PR/SET domain (PRD1-BF1-RIZ/Suvar-Enhancer of Zeste-Trithorax) with histone transferase activity that can catalyze the formation of H3K4Me3 on a H3K4Me2 substrate [109] and a DNA binding domain that includes a tandem array of C2H2 zinc fingers (Figure 9). Several approaches have provided strong evidence that PRDM9 is directly involved in hotspot activity both in mice and humans [105,110,111]. In mice, the *M. musculus domesticus* and *M. musculus molossinus* strains, which have different *Prdm9* alleles, characterized by polymorphisms at positions encoding residues of the zinc finger array involved in interaction with the DNA (zinc finger coordinates −1, +2, +3 and +6), present major genome-wide differences in hotspot and recombination activity [105,108]. Using a zinc finger database [112], the binding sites for *M. m. domesticus* and *M. m. molossinus* PRDM9 can be predicted and were found to be markedly different. The binding site for *M. m. molossinus* PRDM9 is localized at the *Psmb9* and *Hlx1* hotspots (Figure 8) that are active specifically in the presence of this allele. Strikingly, the predicted DNA binding sites for the two major alleles of human PRDM9 (A and B) found in the European population match the 13-mer motif that is enriched at LD-based hotspots [95]. The link between *Prdm9* genotype and CO position was tested by an association study in Hutterites, a population of European ancestry. In this population, a minor *Prdm9* allele (allele I, 2%) encodes a variant predicted to bind to a sequence that is distinct from the one of the A and B variants and that does not match the 13-mer motif. In AA individuals, about 60% of COs fall within LD-based hotspots, whereas in AI individuals only about 18% of COs do so. This dramatic change in CO distribution (without modification of the genetic map length) indicates that the I allele activates a set of hotspots that have left no footprint on genetic diversity. This prediction on binding specificities has been validated by *in vitro* binding assays: the major PRDM9 allele binds with high affinity to a DNA sequence containing the 13-mer motif and the conserved bases in this motif are important for binding [105].

Furthermore, at the DNA2 hotspot, where an imperfect match of the 13-mer motif is found, a polymorphism at one degenerated base is correlated with a variation of hotspot activity [85]. Direct assays at several hotspots in different individuals showed a strong effect of *Prdm9* variations on hotspot activity [113]. Significant effects were observed even at hotspots where consensus binding sites for PRDM9 could not be identified. On the other hand, some PRDM9 variants with little predicted differences in DNA specificity also are associated with significant variations in hotspot activity. Some of these effects could be due to additional *trans* regulators that act (in)dependently from PRDM9, or could result from limitations of the predictability of the DNA binding specificity of PRDM9 zinc fingers. Indeed, although zinc fingers that recognize most of NNN tri-nucleotides have been designed, the prediction of DNA binding for any given zinc finger and, more importantly, for an array of zinc fingers is complex. Changes at positions other than −1 to 6 (*i.e*., the helix residues that interact with the DNA) that may influence affinity as well as cooperation between zinc fingers are not well understood. Indeed, although modular assembly can be used to generate candidate zinc fingers for a given DNA sequence [114], a tool that has been intensely and successfully exploited for genome editing [115], “unknowns” about interactions between zinc fingers complicate the accurate predictions of DNA recognition, particularly in zinc fingers arrays [116]. Furthermore, additional factors could constrain PRDM9 access to its binding site in the genome (DNA methylation, chromatin structure, chromosomal organization). 

### 4.6. What Fraction of Hotspots Depends on *Prdm9*?

The magnitude of the effect of the *Prdm9* genotype on CO distribution observed in the Hutterite population suggests that *Prdm9* plays a major role in hotspot localization and is also compatible with the presence of a fraction of *Prdm9*-independent hotspots [105]. The important difference in LD-based hotspot usage (60% to 18%) between homozygous (AA) and heterozygous (AI) individuals suggests that PRDM9 plays a role in more than the 40% of LD-based hotspots in which the 13-mer motif has been identified. As discussed above, due to the potentially complex PRDM9 binding specificity, this percentage is likely to be an underestimation of the number of PRDM9-dependent hotspots in humans. However, this does not exclude the possibility that, in a minority of hotspots, PRDM9 zinc finger specificity or PRDM9 itself is not involved. Indeed, a few hotspots with comparable activities were detected in two mouse hybrids that carry different *Prdm9* alleles [100]. Additional support for this idea comes from the analysis of *Prdm9^−/−^* spermatocytes and oocytes where a significant level of γ H2AX is detected, indicating that DSBs are formed [109]. In addition, γ H2AX appears to accumulate and the number of DMC1 foci is reduced suggesting a DSB repair defect in *Prdm9^−/−^*. This could either imply that *Prdm9* has a (direct or indirect) role in DSB repair, or that this phenotype is an indirect consequence of an alteration of initiation events. The comparative analysis of gene expression in *Prdm9^−/−^* and wild type mice suggests that *Prdm9* may be required for the expression of genes involved in DSB repair [117]. However one cannot exclude that the observed effects on gene expression could be due to differences in cell composition in *Prdm9^−/−^* and wild type mice. In the absence of PRDM9 a fraction of initiation events may not occur at their correct locations and/or at the proper frequency, thus leading to altered homologous interactions, DSB repair and/or synapsis. 

### 4.7. From PRDM9 Binding to DSB Formation: The Model

**Figure 10:**
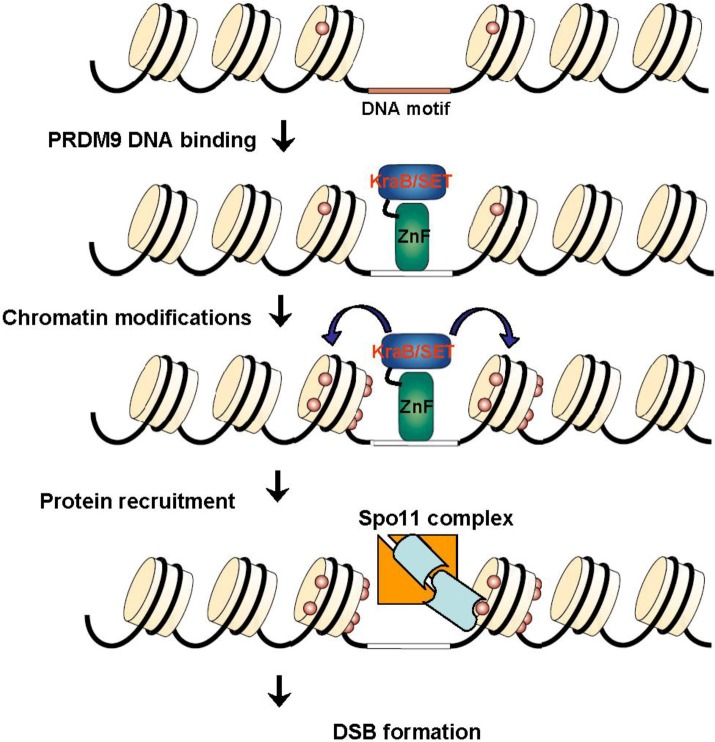
Hotspot specification by PRDM9: PRDM9 binds to a target sequence through its zinc finger domain and brings about H3K4Me3 at adjacent nucleosomes. Additional modifications and proteins recruitment may follow either by interaction with H3K4Me3 and/or with PRDM9, allowing the recruitment of the SPO11 complex and DSB formation.

PRDM9 molecular role in hotspot specification should thus involve binding of the PRDM9 zinc finger domain to a specific DNA sequence. In the context of chromatin marked by H3K4Me2, PRDM9 then induces H3K4Me3 at adjacent nucleosomes. H2B ubiquitination at K123 may or may not be required, depending on the similarities between the modifications mediated by PRDM9 and by methyltransferases that are part of the Compass complex which is active at transcription promoters [118]. Additional proteins may then be recruited through binding affinity for H3K4Me3 and/or PRDM9. Particularly, PRDM9 contains a KRAB motif known to be involved in protein-protein interactions. These modifications eventually allow the recruitment of SPO11 (Figure 10).

### 4.8. Evolutionary Conservation of the Mechanism of Hotspot Specification

It is interesting to compare the features of mammalian hotspots to the chromatin accessibility and the several chromatin modifications that regulate DSB activity in *S. cerevisae* (see [119]). In *S. cerevisiae*, most DSBs are located in promoter regions [18,120] at sites that are enriched in H3K4Me3 independently of the expression levels of adjacent genes [121]. H3K4Me3 is brought about by a unique methyl transferase, Set1, which is recruited by RNApolII. In *set1*∆ strains, DSB activity is strongly reduced and DSBs mostly occur at new sites in the genome that have very low levels of H3K4Me3 in wild type cells [121,122]. However, it is not clear whether H3K4me3 enrichment at hotspots is an evolutionarily conserved feature as it is not detected at the well characterized hotspot ade6-M26 in *S. pombe* [123]. In this species, the ade6-M26 hotspot is a binding site for the transcription factor Atf1-Pcr1, which can recruit chromatin remodelers and modifiers [124,125,126]. Other DNA motifs, which are binding sites for transcription factors, have been shown to stimulate recombination activity in the *S. pombe* genome, suggesting that one or several families of transcription factors could specify initiation sites of meiotic recombination [127,128]. 

### 4.9. Evolution of Initiation Sites in Mammals

A unique feature of the *Prdm9-*dependent mechanism of hotspot specification in mammals is represented by the evolutionary implications that may apply to vertebrates both at the level of hotspot DNA sequences and *Prdm9* gene. One surprising characteristic of hotspots in primates is their poor evolutionary conservation as shown by human/chimpanzees comparisons of LD-based hotspots [129,130]. Myers *et al.* [110] reported that, in chimpanzees, the presence of the 13-mer motif in a repeat background, which is mostly associated with hotspot activity in humans, is not correlated with increased recombination rates. Furthermore, the 13-mer motif goes through a very different evolution in humans and chimpanzees as it is lost at a much higher rate in humans, specifically in the THE1 repeat background. This is an expected property for a DNA motif that stimulates DSB formation in its close proximity in *cis*: Due to the mechanism of DSB repair, the initiating chromatid is the recipient of genetic information and DNA motifs that enhance hotspot activity should thus be replaced by less active alleles during DSB repair. This is known as the hotspot paradox and over generations it should lead to loss of hotspot activity. Reciprocally, the stability of the 13-mer motif in chimpanzees is explained by the fact that the predicted DNA binding specificity of the chimpanzees PRDM9 does not match the 13-mer motif due to several differences at critical residues of many zinc fingers [110]. Whether, the predicted chimpanzees PRDM9 binding motif is enriched at chimpanzees hotspots has not yet been determined. 

This analysis highlights how a few changes in PDRM9 can have large consequences on the global CO distribution. Amazingly, PDRM9 zinc fingers are located in a single exon, have highly similar DNA sequences, and thus constitute a mini-satellite that is highly prone to copy number variations (duplications, deletions) and gene conversion by replication slippage and/or recombination. These features provide a potential answer as to how, despite the hotspot paradox, high recombination activity can be maintained through selection for PRDM9 variants with distinct DNA binding specificity. What selective forces act on *Prdm9* and, in particular, how a progressive decrease of PRDM9 binding sites would affect recombination activity and how this could affect fitness, is yet unclear. In any case, the phylogenic analysis of *Prdm9* among vertebrates has shown that PRDM9 zinc finger repeats are undergoing concerted evolution and that *Prdm9* is under strong positive selection [131,132]. 

## 5. Conclusions

SPO11-dependent meiotic DSBs play an essential role in meiosis by initiating the process that leads to CO formation and thus ensuring the proper segregation of chromosomes at meiosis I. However, the number of DSBs largely exceeds the number of COs in mammals, with an average of 10–20 times more DSBs than COs [11]. Molecular analysis at several hotspots both in humans and mice indicates that at least a fraction of the DSBs that do not give rise to COs are repaired on the homolog and generate NCOs [79,80,81,82,83,84,90]. One potential role for DSBs may be to promote homology search and pairing between homologs. Interestingly, in *C. elegans* and *D. melanogaster*, where homologs can pair independently from recombination through alternative pathways, the total number of DSBs is about 12 [133] and 20–24 respectively [134], thus much lower than the several hundred DSBs observed in mammals. This also implies that mammals must therefore deal with a major threat to genome integrity during meiosis. Although the meiotic nucleus and chromosomes are organized in such a way as to appropriately repair these DSBs, rare unscheduled events may occur. In particular, DSBs can be repaired by interaction with a non-allelic sequence, leading to deletion or duplication in the case of interaction with an intra-chromosomal sequence, or to translocation in the case of inter‑chromosomal sequences [135]. Some disorders, due to such genome rearrangements [136], do occur during meiosis [137]. More importantly, PRDM9 binding sites have been identified near regions involved in these rearrangements, and the *Prdm9* genotype influences the occurrence of these events [113]. 

Recent progress in the understanding of the mechanism of initiation of meiotic recombination in mammals opens many new questions and hypotheses that are expected to provide important clues about this phenomenon, and also on how its deregulation could contribute to genomic disorders and sterility. The molecular mechanisms of DSB formation is likely to involve complex interactions between catalytic activities and proteins that play structural roles to coordinate DNA and chromosome events, in order to establish a regulation that could sense a DNA event and operate at the level of the chromosome. These features have been described in other organisms with the role of components of chromosome axis for DSB formation, such as Hop1 in *S. cerevisiae* [4] and Condensins in *C. elegans* [133]. In addition, initiation sites are expected to involve a sophisticated combination of epigenetic marks of which most components are waiting to be discovered.
